# Impact of Feeding Fermented Palm Kernel Cake and High Dietary Fat on Nutrient Digestibility, Enzyme Activity, Intestinal Morphology and Intestinal Nutrient Transporters mRNA Expression in Broiler Chickens under Hot and Humid Conditions

**DOI:** 10.3390/ani12070882

**Published:** 2022-03-31

**Authors:** Ali Hanafiah Hakim, Idrus Zulkifli, Abdoreza Soleimani Farjam, Elmutaz Atta Awad, Suriyah Kumari Ramiah

**Affiliations:** 1 Tropical Agriculture and Food Security, Universiti Putra Malaysia, UPM Serdang 43400, Selangor, Malaysia; hakim.ma@umk.edu.my (A.H.H.); absf@novozymes.com (A.S.F.); motazata83@gmail.com (E.A.A.); s_kumari@upm.edu.my (S.K.R.); 2 Department of Agricultural Science, Faculty of Agro-Based Industry, Universiti Malaysia Kelantan, Jeli Campus, Jeli 17600, Kelantan, Malaysia; 3 Department of Animal Science, Faculty of Agriculture, Universiti Putra Malaysia, UPM Serdang 43400, Selangor, Malaysia; 4 Novozymes Malaysia Sdn Bhd, Technology Park Malaysia, Kuala Lumpur 57000, Wilayah Persekutuan, Malaysia; 5 Department of Poultry Production, Faculty of Animal Production, University of Khartoum, Khartoum 13314, Sudan

**Keywords:** broiler chicken, palm kernel cake, alternative feed, fermentation, palm oil

## Abstract

**Simple Summary:**

The use of palm kernel cake (PKC) as an alternative ingredient in poultry diets is limited due to its high fibre content, which affects the growth of chicken. The fermentation of PKC with lactic acid bacteria has been shown to improve the broiler’s growth performance even at an increasing level of PKC inclusion. On the other hand, a higher level of PKC/fermented PKC (LPKC) inclusion in the broiler diet has always been incorporated with a higher level of dietary fat supplementation. However, there is little information on the effect of feeding PKC/LPKC based diets and oil supplementation on the digestion and absorption processes. We observed the response of broiler chickens to a fermented PKC-based diet at different levels of dietary fats, under hot and humid conditions. Higher oil inclusion in a PKC/LPKC diet is necessary to ensure better nutrient digestibility in chickens via improved digestive function. This finding will enable better utilisation of the agricultural by-product, and a more optimum feed formulation, especially in hot and humid tropical regions.

**Abstract:**

The study aimed at determining the ileal nutrient digestibility, digestive enzyme activity, intestinal morphology, and nutrient transporters mRNA expressions in broiler chickens fed with fermented PKC (LPKC) based diets with different levels of fat supplementation under hot and humid conditions. From day 22 to 35, broiler chickens were randomly fed with either (1) 20% LPKC-based diet with 5% palm oil, (2) 20% LPKC based diet with 9.5% palm oil, (3) 20% PKC-based diet with 5% palm oil or (4) 20% PKC-based diet with 9.5% palm oil. Feeding LPKC and PKC diets at the finisher phase have not affected the nutrient’s digestibility, but a higher level of oil supplementation does. This was seconded by changes in the digestive enzyme activity, villus height, and mRNA expression of nutrient transporters in the higher level of oil-supplemented diets fed chickens. In conclusion, the inclusion of oil at 9.5% in a 20% LPKC/PKC-based diet is necessary to ensure better nutrient digestibility in chickens via improved digestive function, especially in hot and humid tropical regions.

## 1. Introduction

The dependency of the poultry industry in most developing countries on imported feed ingredients has been a significant threat to its sustainability. Particularly in Malaysia and other palm oil-producing countries, the abundance of palm kernel cake (PKC) production as one of the by-products offers a possible solution to replace or partially replace the inclusion of corn and soybean meal as the main energy and protein source in the poultry diet. Despite having a moderate amount of energy and crude protein (CP), PKC utilization in the poultry diet has been limited by several factors such as high crude fibre and indigestible non-starch polysaccharides (NSP), low essential amino acids (AA) concentration, poor nutrient digestibility and less palatability [1,2,3].

Higher levels of dietary fat have always accompanied the inclusion of a higher level of PKC in the poultry diet to compensate for poor metabolizable energy (ME) supplied by PKC. For instance, Saenphoom, et al. [4] doubled the palm oil inclusion between a PKC-free diet and a 20% PKC diet, while Alshelmani, et al. [5] increased palm oil inclusion by nearly 3-fold between a PKC-free diet and a 15% PKC diet. Zulkifli, et al. [6] found that adding a higher palm oil level to a PKM-based diet may improve the tolerance of heat-stressed broilers to the depressive effects of fibre in PKM. Our laboratory’s more recent evidence has shown better growth performance in broilers fed with the PKC diet at 9.5% palm oil supplementation than in the PKC diet with 5% palm oil [7]. Earlier studies demonstrated the ability of fibre to alter the intestinal environment and reduce the feed retention time [8,9,10], while the inclusion of dietary fat showed the opposite effects [11], however, both may affect the nutrient utilization and growth response [12,13]. Nevertheless, the mechanisms of dietary fat in improving nutrient utilization in PKC-containing diets are not clearly understood.

Alshelmani, et al. [5] suggested that the presence of high levels of neutral detergent fibre (NDF), acid detergent fibre (ADF), insoluble fibres, and NSPs in the PKC may depress the apparent ileal digestibility of nutrients. Similarly, other NSP-containing materials such as wheat, rye, and barley have been associated with low nutrient digestibility [14,15], disruption of digestive enzyme mechanism [16,17], lower nutrient transporter expression [18], and intervention with the intestinal environment [8,9,10,13]. Several methods have been introduced as potential means for better NSP degradation of PKC, and to improve nutrient availability [1,19,20,21,22]. We have previously reported improvement in nutritional values, metabolisable energy, and digestibility of CP and AA in a *Weisella confusa* fermented PKC (LPKC) compared to the untreated PKC for broilers under hot and humid environmental conditions [23]. 

In the small intestine, digestive enzymes and transporter proteins play an essential role in the digestion and absorption of dietary nutrients, respectively [24]. Digestive enzymes are responsible for the hydrolysis of dietary nutrients along the small intestines and thus the efficiency of absorption of the digested product [25]. The digestive enzymes activities in chickens are well-documented in many aspects, including nutrition [26,27,28], feeding regimen [29], age [30], and health status [31]. Feeding a diet containing hull polysaccharides was detrimental to digestive trypsin and lipase activities in response to forming an enzyme-fibre complex in the gut, which may decrease the availability of enzymes for the substrates [32]. On the other hand, Jin, et al. [27] observed an elevation of intestinal amylase activity upon supplementation of the adherent Lactobacillus cultures to chickens, either as a single strain of L. acidophilus or as a mixture of 12 Lactobacillus strains. Hence, the digestive enzyme may interact differently with a high fibre diet and lactic acid bacteria fermented/supplemented diet.

Amino acids are transported across the enterocytes in di- or tripeptides by H+−dependent peptide transporter, PepT1, or as free amino acids by various amino acids transporter. The transport of carbohydrates is facilitated in various forms: glucose and galactose by Na+−dependent glucose transporter, SGLT1, and SGLT5, or in the form of fructose transported by GLUT5. In contrast, lipids are transported as fatty acids by adhering to fatty acids binding protein FABP1. Research has shown that the expression of SGLT1 and PepT1, and a few other transporters are highly associated with nutrient absorption capacity [33,34,35]. Few authors reported the role of nutrient transporter gene expression and its association with lower growth rates in chickens exposed to various stressors [36,37]. Sun, et al. [38] reported that heat stress adversely affected the jejunal glucose (GLUT-2) and lipid (CD36 and FABP1) transporters but not the amino acids transporters, which were accompanied by reduced weight gain and feed intake in chickens. However, little is known regarding the influence of diet composition, especially fibre/NSP, on the expression of nutrient transporters in poultry.

We hypothesised that the combination of low fibre in LPKC and high dietary fat inclusion may improve nutrient utilization and digestive response; therefore, LPKC could be a potential alternative feedstuff under hot and humid conditions in tropical regions. Thus, this study aimed at determining the ileal nutrient digestibility, digestive enzyme activity, intestinal morphology, and nutrient transporters mRNA expressions in broiler chickens fed with fermented PKC-based diets with different levels of fat supplementation.

## 2. Methodology

### 2.1. Birds and Management

The experimental procedure was validated by the institutional animal care and use committee (IACUC) of the Universiti Putra Malaysia (UPM/IACUC/AUP-R021/2018). A total of 240 one-day-old Cobb 500 male broiler chicks were acquired from a local hatchery. Upon arrival (day 1), chicks were weighed and randomly assigned to 24 battery cages (240 × 120 × 45 cm, length × width × height) with wire floors in a conventional open-sided house with ten chicks per cage. The temperature and relative humidity ranged from 24 °C to 36 °C, and 60–88%, respectively (Table 1). On day 7 and 21, birds were vaccinated via intraocular route against Newcastle disease (ND) and Infectious bronchitis (IB). Water was always accessible and birds were fed ad libitum.

### 2.2. Diets and Experimental Design

Two types of PKC (PKC and LPKC) and two degrees of oil inclusion design (5% and 9.5%) were included in the treatments, which were arranged in a 2 × 2 factorial arrangement. PKC was purchased from a commercial kernel oil extraction factory in Klang, Selangor, Malaysia, and crushed to a consistent size of around 3 mm prior to treatment. LPKC was produced by inoculating lactic acid bacteria of the strain *Weisella confusa* SR-17b with PKC using a solid-state fermentation method, as described by Tan [39].

From d1 to 21, all chicks were fed with a standard broiler starter ration (Table 2). Then, equal numbers of birds were randomly assigned to one of four dietary treatments from d 22 to 35: 20% LPKC with 5% Palm Oil (LPKC-LO), 20% LPKC with 9.5% Palm Oil (LPKC-HI), 20% PKC with 5% Palm Oil (PKC-LO) and 20% PKC with 9.5% Palm Oil (PKC-HI) (Table 2). Each dietary group was made into six replicates in a complete randomised design. All diets were isocaloric and isonitrogenous, meeting or exceeding the nutrient requirements recommended for Cobb 500 broilers (Cobb-Vantress Inc., Siloam Springs, AR, USA). The PKC and LPKC diets were formulated based on nutrient and digestible nutrient compositions which were pre-determined in our laboratory [23].

### 2.3. Samples and Data Collection

On day 32, all birds were offered the assay diets containing 0.5% TiO2 and these were offered for four consecutive days. On day 35, 2 birds per cage were randomly selected and slaughtered humanely by following the halal slaughtering procedure [40]. A homogenous duodenum and jejunum digesta sample was collected for enzyme activity determination by gently squeezing the two intestinal parts. To avoid mixing digesta samples with blood and excess tissue, care was taken to minimise interference with the enzyme assay. The digesta samples were immediately frozen in liquid nitrogen and stored at −80 °C until used. The ileal contents were collected for nutrient digestibility analysis by gentle flushing with distilled water and pooled within each cage. The samples were freeze-dried and stored in airtight containers at −20 °C. The tissue sample section (5 cm) was cut out from the middle part of the duodenum, jejunum, and ileum, gently flushed, and fixed in 10% formalin solution for later histology measurements. A portion of the jejunum tissue sample (5 cm) was also cut out and gently flushed with normal saline. It was immediately frozen in liquid nitrogen and stored at −80 °C for mRNA analysis.

### 2.4. Digestive Enzyme Activity

Digestive enzyme activities of amylase activity, lipase activity, and trypsin activity were determined using the commercial Amylase Activity Assay Kit (Sigma-Aldrich, MAK009, St. Louis, MO, USA), Lipase Activity Assay Kit (Sigma-Aldrich, MAK046), and Trypsin Activity Assay Kit (Sigma-Aldrich, MAK290), respectively, according to the manufacturer’s instructions (Merck KGaA, Darmstadt, Germany).

### 2.5. Nutrient Digestibility

All diets and ileal digesta samples were analysed for their DM, CP, ether extract (EE), ash, gross energy (GE), and titanium concentrations. Proximate analysis for DM, nitrogen content (N), EE, and ash was determined following the procedure of AOAC [41]. The CP was calculated as N × 6.25. The GE was analysed using a bomb calorimeter (IKA2000©, IKA-Werke GmbH & Co. KG, Staufen, Germany). For titanium determination, the samples were furnaced at 580 °C for 13 h to burn all organic material, and the remaining minerals were digested (using 7.4 M sulphuric acid) to release titanium dioxide content, which was determined using a spectrophotometer (SPECORD PLUS©, Analytic Jena AG, Jena, Germany) at a wavelength of 410 nm [42].

The apparent ileal digestibility (AID) of each nutrient was determined following the calculations described by Awad, et al. [43]:

$${\mathrm{AID}}_{\mathrm{diet}}=100\;-\;(\frac{{\mathrm{Nutrient}}_{\mathrm{digesta}}}{{\mathrm{Nutrient}}_{\mathrm{diet}}}\times \frac{{\mathrm{Ti}}_{\mathrm{diet}}}{{\mathrm{Ti}}_{\mathrm{digesta}}}\times 100)$$

### 2.6. Intestinal Histomorphology

Intestinal morphology analysis was carried out following the method described by Awad [44]. Formalin-fixed intestinal samples were dehydrated and then embedded in paraffin wax. Sample sections of 5-μm thicknesses were placed on glass slides, heated at 55 °C, and stained with hematoxylin and eosin. The stained sections were examined using a light microscope to measure the villi height and crypt depth (10 villi and 10 crypts per section). The villi height was measured from the apex of the villi to the villi-crypt junction, while the crypt depth was referred to as the depth of the invagination between adjacent villi.

### 2.7. mRNA Analysis of Nutrient Transporter

The mRNA analysis of the nutrient transporter was carried out following the method described by Izuddin, et al. [45]. The total RNA was extracted from jejunum tissues (6 biological replicates per treatment, *n* = 6) using the RNeasy^®^ Mini Kit (Qiagen, Hilden, Germany), following the manufacturer’s protocol. Total RNA concentration and purity were measured using Nanodrop 2000 spectrophotometer (Thermo Scientific, Wilmington, DE, USA), and only high purity samples were chosen for the reverse transcription process. According to the manufacturer’s instruction, approximately 1000 ng/μL of purified RNA was converted into complementary DNA (cDNA) using Quantitect^®^ Reverse Transcription Kit (Qiagen, Hilden, Germany). Bio-Rad CFX96 Real-time PCR system (Bio-Rad Laboratories, Hercules, CA, USA) was used to perform the real-time qPCR step. The forward and reverse sequence and product size of the target and reference genes were presented in Table 3.

The PCR reaction was carried out using QuantiNova™ SYBR Green PCR kit (Qiagen, Hilden, Germany) containing a total of 20 μL PCR master mix consisting of 10 μL 2× SYBR Green PCR Master Mix, 1 μL forward primer, 1 μL reverse primer, 7 μL RNase-free water and 1 μL template cDNA. The qPCR cycling condition consists of initial heat activation at 95 °C for 5 min, followed by 40 cycles of denaturation at 95 °C for 10 s, annealing at 56 °C for SGLT1, PepT1, and EAAT3 genes, 58 °C for SGLT5, rBAT, and FABP1 genes, and 60 °C for GAPDH and B-actin, and followed with 30 s of extension at 72 °C. The optimum annealing temperature of target and reference genes was determined by Bio-Rad CFX96 Real-time PCR System (Bio-Rad Laboratories, Hercules, CA, USA). The melting curve analysis was performed at the end of the amplification cycle to confirm the specificity of amplification. The relative expression of the gene was measured according to Livak’s method of 2^−ΔΔCt^ (ΔΔCt = ΔCt treated sample − ΔCt calibrator sample) as described by Livak and Schmittgen [46]. For the internal standard (housekeeping gene), GAPDH was used to standardise the expression. A 5-fold serial dilution of cDNA determined the efficiency of amplification of target and housekeeping genes as a standard curve.

### 2.8. Statistical Analyses

All data were subjected to 2-way ANOVA using the SAS software’s GLM procedure [47] (SAS Institute Inc., Cary, NC, USA). The statistical model tested the effects of types of PKC, level of oil, and their interactions as the main effects. Means were separated using Duncan’s multiple range test. Statistical significance was considered as *p* < 0.05.

## 3. Results

### 3.1. Nutrient Digestibility

The results of the apparent ileal nutrient digestibility of broilers fed LPKC and PKC based diets at different levels of oils are presented in Table 4. There were no significant type of PKC × level of oil interactions for nutrient digestibility. Type of PKC had no significant effect on the digestibility of DM (*p =* 0.4510), GE (*p =* 0.1091), CP (*p =* 0.2056), EE (*p =* 0.0934), and ash (*p =* 0.4572). However, feeding diets with 9.5% oil significantly increased the digestibility of DM (*p <* 0.0200), GE (*p <* 0.0065), CP (*p <* 0. 0001), and ash (*p <* 0.0175) as compared to diets supplemented with 5% oil, while no difference (*p =* 0.1284) was noted for EE digestibility.

### 3.2. Intestinal Digestive Enzyme Activity

Results of intestinal digestive enzyme activities are shown in Table 5. No significant type of PKC x level of oil interaction was noted for enzyme activity. Chickens fed PKC and LPKC diets had similar intestinal amylase (*p* = 0.0586), protease (*p* = 0.9191), and lipase (*p =* 0.4974) activities. Diets with 9.5% oil significantly decreased (*p* < 0.0001) the intestinal amylase activity compared to the group fed 5% oil. The level of oil had a negligible influence on the intestinal protease (*p =* 0.8876) and lipase (*p =* 0.1198) activities.

### 3.3. Intestinal Morphology Indices

There were no significant type of PKC x level of oil interactions for intestinal villi height and crypt depth (Table 6). Feeding LPKC diets significantly increased the duodenal villi height (*p =* 0.0003) and the crypt depth of jejunum (*p =* 0.0103) and ileum (*p =* 0.0193) compared to the PKC- fed chickens. The duodenal crypt depth (*p =* 0.2482), and the villi height of jejunum (*p =* 0.9274) and ileum (*p =* 0.9580) were not significantly affected by the type of PKC. A diet with 9.5% oil inclusion significantly (*p* < 0.0001) increased the height of duodenal villi compared to those fed the 5% oil diet. The villi height of jejunum (*p* = 0.2634) and ileum (*p* = 0.2279), and crypt depth of duodenum (*p* = 0.5348), jejunum (*p* = 0.8418) and ileum (*p* = 0.9883) were not affected by the level of oil.

### 3.4. Gene Expressions of Nutrient Transporters Analysis

Table 7 shows the effects of PKC type and oil level on gene expressions of nutrient transporters. No significant type of PKC × level of oil interaction was noted for the mRNA expression of nutrient transporters. Birds fed the LPKC diets exhibited higher expression of r-BAT (*p* < 0.0001) and EAAT3 (*p* < 0.0001), and lower expression of SGLT-5 (*p* < 0.0001) and FABP1 (*p* < 0.0001) than their counterparts fed the PKC diet. Feeding a diet with 9.5% oil upregulated the expression of SGLT-1 (*p* < 0.0001), SGLT-5 (*p* < 0.0001), Pep-T1 (*p* < 0.0001), r-BAT (*p* < 0.0001), EAAT3 (*p* < 0.0001) and FABP1 (*p* = 0.0159) when compared to the group provided with the 5% oil diet.

## 4. Discussion

PKC constituted up to 68% of fibre, of which 78% were in the form of linear, insoluble, and highly crystalline β-mannans, with some galactose substitution, 12% cellulose, 3% (4-O-methyl)- glucuronoxylans, and 3% arabinoglycans [48]. Upon fermentation or inoculation with a functioning inoculum, a major part of fibre and NSP in PKC may undergo enzymatic hydrolysis, which is then reduced to various monosaccharides, subsequently decreasing the NSP concentration [19,22,49]. Such changes in the feed may contribute to the improved digestibility of nutrients in NSP-containing feed due to the alteration in intestinal viscosity and environment [5,50].

In the current study, both PKC and LPKC diets showed similar GE, DM, CP, EE, and ash digestibility (Table 4). Conversely, Alshelmani, et al. [5] noted that LAB-fermented PKC had better DM and CP digestibility than the untreated PKC diet. Navidshad, et al. [51] also found that birds fed with an enzyme-treated PKC diet had higher digestibility of DM, CP, and EE compared to groups fed with an untreated PKC diet. The inability of LPKC diets to express better nutrient digestibility could be attributed to the similar fibre content (CF and NDF) of LPKC and PKC diets (Table 2). Although LAB fermentation of PKC resulted in a significant reduction of NDF concentration by 5% [23], this reduction may not be substantial enough to improve the digestibility when LPKC was incorporated into the diet.

The present findings showed that feeding higher oil levels increased the digestibility of DM, GE, CP, EE, and ash (Table 4). Higher dietary fat has been reported to increase the nutrient retention time in broiler chickens, which may improve nutrient digestion and absorption [11,12,13]. Honda, et al. [52] compared nutrient digestibility at different sites of the intestines in broilers fed different levels of tallow. The authors concluded that higher dietary fat influenced feed passage time at the middle part of the ileum but had a negligible effect on nutrient digestibility at the ileum and rectum. This may suggest that modulating the feed passage time in a high viscous diet such as PKC is essential for adequate substrate-enzyme interaction at a specific digestion site.

Mathiavanan, et al. [53] fed broilers with fermented soybean meal for six weeks, and did not observe any influence on the small intestines’ digestive amylase and trypsin activities. Similarly, in the current study, neither PKC nor LPKC significantly affected the intestinal enzyme activities of 35-day old broilers (Table 5). Feng, et al. [54] reported that intestinal trypsin, lipase, and protease activities were only affected by the feeding of fermented soybean meal during the starter phase. The effect of age on the activities of digestive enzymes was also indicated by Luo, et al. [55]. The authors demonstrated that xylanase supplementation in a wheat-based diet did not alter the digestive enzyme activities in the small intestines of broiler chickens at 42 days of age. Almirall, et al. [31] suggested that older birds were better able to cope with intestinal viscosity changes than younger chicks. This could be attributed to incipient microflora development in younger birds’ intestines, which is essential in reducing the anti-nutritional effects of NSPs [56]. Thus, the lack of fermented feed influencing intestinal enzyme activities in older chickens was expected.

We found that diets with higher oil levels reduced the intestinal amylase activity in broilers irrespective of PKC type (Table 5). This finding concurs with Almirall, et al. [30] and Luo, et al. [55] that the digestive enzymes of older birds were more affected by the presence of dietary fat instead of fibre. Increased amylase activity has been linked with feed-restricted birds and low-feed intake birds, which acts as a compensatory mechanism for intestinal adaptation to low energy intake [29,57,58]. Higher dietary fat supplementation in the diet of layer hens has shown longer nutrient retention time, thus allowing better energy utilization [11]. Thus, the lower amylase activities in high oil supplemented fed diet-fed birds may have resulted from improved energy intake due to longer feed retention time. On the contrary, Hulan and Bird [59] indicated that reduced dietary fat increased intestinal amylase activity in chickens. According to the authors, the noted increased amylase activity could be attributed to higher carbohydrate intake in the lower-fat diet group because the lower and higher fat diets were not isocaloric, which may result in inconsistent findings with the current study.

The size and height of the intestinal villi and crypt are essential for growth by holding the primary site for nutrient absorption and acting as a villi factory for tissue turnover and production, respectively [60,61,62]. In this study, feeding LPKC diets increased the duodenal villi height and the crypt depth at the jejunum and ileum compared to PKC diets (Table 6). The degradation of PKC through various treatments may increase the concentration of mannan oligosaccharides (MOS) which carry the prebiotic effects [49,63,64]. Prebiotics, such as MOS, have been shown to increase villus height and surface area [65,66] via greater short-chain fatty acids (SCFA) production from prebiotic fermentation [67]. Thus, it can be assumed that LPKC contains a significant amount of prebiotic compared to untreated PKC, which may increase the SCFA production and, subsequently, the intestinal morphology of chickens. Saenphoom, et al. [4] reported increased villi heights and crypt depth in the duodenum and jejunum of broilers fed enzyme-treated PKC diet compared to those provided with an untreated PKC diet to the decreased fibre content upon enzyme treatment. On the other hand, Alshelmani, et al. [5] did not observe any significant effects on small intestines’ villi heights and crypt depths when broilers were fed PKC and fermented PKC based diets at a 15% level of dietary inclusion. The lower inclusion level of PKC and fermented PKC by Alshelmani, et al. [5] compared to the present study (15% vs. 20%) may have accounted for the inconsistent findings.

The present findings indicated that diets with 9.5% oil resulted in longer duodenal villi than the 5% oil diet (Table 6). Higher levels of dietary fat feeding in chickens were found to lengthen the feed passage rate [11], which was linked to reduced digesta viscosity [30,68]. The changes in digesta viscosity have been reported to increase SCFA production [8,9] and may subsequently increase the intestinal villus height and surface area [65,66,67]. These statements may suggest that a higher level of oil feeding to broilers in the current study may indirectly contribute to the greater production of SCFA, thus resulting in increased villi height. The literature regarding the influence of dietary fat levels on intestinal morphology in poultry is limited. Incharoen, et al. [69] reported that the duodenal villi area but not height was reduced in chickens fed lower fat diets than controls (2.9% vs. 4.9%). Schiavone, et al. [70] fed broilers with different levels of black soldier fly larvae fat (3.4% vs. 6.9%) and noted no significant changes in intestinal morphology. There is no clear explanation for these inconsistent results; however, it could be pointed to the wide range of dietary fat levels in the current study (4.5%) as compared to the study reported by Incharoen, et al. [69] (2%) and Schiavone, et al. [70] (3.5%). Besides, variation in dietary fat sources in the current study (palm oil) and Schiavone, et al. [70] (black soldier fly larvae fat) may influence intestinal morphology. Sharifi, et al. [71] reported that diets with soybean oil but not free fatty acids increased duodenal villi length in broilers. Fats rich in lauric acid such as coconut oil may also improve gut development and nutrient absorption, thus enhancing animal growth [72,73].

Table 7 showed that feeding LPKC diets upregulated the expression of glucose (SGLT-5) and amino acids (r-BAT and EAAT3) transporter genes. This finding agrees with Al-Khalaifah, et al. [74], where feeding of microbial fermented and enzymatically treated dried brewer’s grains in broiler chicken have upregulated the expression of all glucose and amino acids transporter. Besides, supplementation of low energy diets with an exogenous enzyme in broiler diets has promoted the absorption of the micronutrients via upregulation of GLUT-2 and Pep-T1 expressions [75]. Ramiah, et al. [76] reported less shell PKC and enzyme-treated PKC feeding has upregulated the protein kinase C zeta (PRKCZ) gene expression, which plays an essential role in the insulin signalling pathway, and the mechanisms of glucose storage and uptake, protein synthesis and regulation of lipid synthesis. On the other hand, higher inclusion levels of oils in the PKC/LPKC diets have upregulated all transporters’ expressions compared to those with lower oil content (Table 7). No clear explanation could be provided for such events. However, these could be indirectly caused by the physiological changes in the intestinal environment, as reducing insoluble mannan NSP in PKC and increasing the level of oil would reduce the digesta viscosity and increase feed passage time [11,30,68]. This may affect the nutrient-to-transporter adhesion. Association between altered viscosity and increased Pep-T1 and SGLT-1 expression was also postulated with chickens fed with xylanase, and exogenous enzyme (Avizyme^®^) supplemented diets [31,75]. As the expression of nutrient transporter genes were positively correlated with the level of available nutrient molecules [77], it can be assumed that reduced viscosity in the LPKC containing diet and the higher oil level diet have improved the diet composition, thus triggering the changes in expression of nutrient transporter genes [75].

Overall, feeding LPKC or PKC diets at the finisher phase did not affect the nutrients’ digestibility and digestive enzyme activity, despite better villus development and higher glucose and amino acids transporter expression in LPKC fed chicken. This finding concurs with Chen, et al. [78] and Saenphoom, et al. [4], where feeding fermented PKC showed no improvement in growth performance in broiler chickens despite significant NSP degradation and release of mannan oligo- and monosaccharides upon the fermentation process. Such responses could be attributed to the lack of assimilation capability by mannose sugar, which is mostly released from NSP of PKC degradation, compared to glucose [79]. On the other hand, a higher level of oil supplementation in the diet has shown better nutrient digestibility, irrespective of PKC type. This was supported by changes in the digestive enzyme activity, villus height, and mRNA expression of nutrient transporters in the higher level of oil-supplemented diet-fed chickens. This agrees with Zulkifli, et al. [6], where the inclusion of a higher level of dietary fat was suggested to alleviate the adverse effects of high fibrous diets. The mechanism of nutrient utilization by the presence of higher oil level in PKC/LPKC diets require further investigation.

## 5. Conclusions

In conclusion, feeding LAB fermented PKC to broiler chickens has improved the intestinal development and expression of glucose and amino acid transporter but had no effects on nutrient digestibility and digestive enzyme activity. Regardless of the type of PKC, diets with 9.5% oil had better nutrient digestibility, digestive enzyme activity, intestinal development, and overall nutrient transporter genes expression. Thus, based on these findings, finisher diets with 20% LPKC and 9.5% oil can be fed to broiler chickens under hot and humid tropical conditions, with better effects in mitigating the adverse impacts of heat stress on broiler’s growth.

## Figures and Tables

**Table 1:** Profile of environmental temperature and humidity during the experimental period.

Experimental Period	Temperature, °C		Relative Humidity, %	
	Minimum	Maximum	Minimum	Maximum
Week 1	24	33	58	88
Week 2	23	33	64	90
Week 3	23	31	68	95
Week 4	24	34	62	90
Week 5	25	36	62	88

**Table 2:** Feed ingredients and chemical composition of the starter and finisher broiler diets.

Ingredient (%, as Fed Basis)	Diets				
	Starter (Day 1–21)	Finisher (Day 22–35)			
		LPKC-LO	LPKC-HI	PKC-LO	PKC-HI
Corn	51.26	45.53	39.63	42.91	35.77
Corn gluten meal	-	6.67	-	7.96	-
Soybean meal	40.00	7.93	27.56	-	19.18
Fullfat soybean meal	-	11.22	-	20.43	12.23
Fermented PKC	-	20.00	20.00	-	-
PKC	-	-	-	20.00	20.00
Palm oil	5.00	5.00	9.50	5.00	9.50
L-Lysine	0.07	0.41	0.13	0.45	0.15
DL-Methionine	0.28	0.21	0.25	0.21	0.27
L-Threonine	-	0.08	0.06	0.09	0.08
DCP	1.82	1.53	1.48	1.53	1.47
Limestone	0.92	0.65	0.65	0.65	0.63
Salt	0.50	0.35	0.35	0.35	0.35
Vitamin Premix	0.05	0.05	0.05	0.05	0.05
Mineral Premix	0.10	0.10	0.10	0.10	0.10
Choline Chloride	-	0.08	0.05	0.07	0.03
Antioxidant	0.10	0.10	0.10	0.10	0.10
Toxin binder	0.10	0.10	0.10	0.10	0.10
Nutrients composition calculated (%, unless stated otherwise)					
ME (kcal/kg)	3065.00	3177.00	3177.00	3177.00	3177.00
CP	22.36	19.67	19.67	19.67	19.67
EE	7.52	9.98	12.36	11.36	14.21
CF	3.01	4.36	4.55	5.46	5.77
NDF	11.62	22.86	22.58	24.01	23.93
Dig Lys	1.18	0.96	0.95	0.96	0.96
Dig Met + Cys	0.88	0.75	0.74	0.75	0.75
Dig Thr	0.77	0.65	0.65	0.65	0.66

PKC, palm kernel cake; DCP, dicalcium phosphate; ME, metabolizable energy; CP, crude protein; EE, ether extract; CF, crude fiber; NDF, neutral detergent fiber; Dig Lys, digestible lysine; Dig Met + Cys, digestible methionine + cysteine; Dig Thr, digestible threonine. LPKCLO, 20% fermented palm kernel cake with 5% palm oil; LPKCHI, 20% fermented palm kernel cake with 9.5% palm oil; PKCLO, 20% palm kernel cake with 5% palm oil; PKCHO, 20% palm kernel cake with 9.5% palm oil. Premixed administered vitamins per kilogram of the diet: vitamin A (retinyl acetate), 8000 IU; vitamin D3 (cholecalciferol), 1000 IU; vitamin E (DL-α-tocopherol), 30.0 IU; vitamin K3 (menadione dimethylpyrimidinol, 2.50 mg; vitamin B1, 2.00 mg; vitamin B2, 5.00 mg; vitamin B6, 2.00 mg; vitamin B12, 0.01 mg; niacin, 30.0 mg; d-biotin, 0.045 mg; vitamin C, 50.0 mg; d-pantothenate, 8.00 mg, folic acid, 0.500 mg. Premixed administered minerals per kilogram of the diet: Mn, 70.0 mg; Fe, 35.0 mg; Zn, 70.0 mg; Cu, 8.00 mg; I, 1.00 mg, Se, 0.250 mg; Co, 0.200 mg.

**Table 3:** Primer sequence for gene expression analysis.

Gene	Primer Sequences	Product Size (bp)	Reference
FABP1	F: ACTGGCTCCAAAGAATGACCAATG	163	(Sun et al., 2015)
	R: TGTCTCCGTTGAGTTCGGTCAC		
r-BAT	F: CTTCGCAACAGTGAGCTACCCATA	109	(Sun et al., 2015)
	R: TAAAGACGCTGTCTAACCCATCCAA		
EAAT3	F: TGCTGCTTTGGATTCCAGTGT	79	(Ebrahimi et al., 2015)
	R: AGCAATGACTGTAGTGCAGAAGTAATATATG		
PepT-1	F: CCCCTGAGGAGGATCACTGTT	205	(Ebrahimi et al., 2015)
	R: CAAAAGAGCAGCAGCAACGA		
SGLT-1	F: TGTCTCTCTGGCAAGAACATGTC	229	(Ebrahimi et al., 2015)
	R: GGGCAAGAGCTTCAGGTATCC		
SGLT-5	F: ATACCCAAGGTAATAGTCCCAAAC	75	(Ebrahimi et al., 2015)
	R: TGGGTCCCTGAACAAATGAAA		
GAPDH	F: GCCGTCCTCTCTGGCAAAG	128	(Ebrahimi et al., 2015)
	R: TGTAAACCATGTAGTTCAGATCGATGA		

SGLT-1, sodium-glucose transporter 1; SGLT-5, sodium-glucose transporter 5; Pep-T1, H+/peptide transporter; r-BAT, protein related to b^0,+^ amino acid transport system; EAAT3, excitatory amino acid transporter 3; FABP1, fatty acid binding protein 1; GAPDH, glyceraldehyde phosphate dehydrogenase.

**Table 4:** Apparent ileal digestibility (%) of nutrients of broilers fed LPKC and PKC-based diets at different levels of oil (Mean ± SE).

Diet	Nutrient, %				
	DM	GE	CP	EE	Ash
Type of PKC					
PKC	59.58 ± 1.10	60.87 ± 0.94	75.31 ± 0.55	89.38 ± 0.72	37.56 ± 1.79
LPKC	61.16 ± 0.95	62.79 ± 0.92	75.98 ± 0.72	87.36 ± 0.91	39.24 ± 1.67
Level of Oil					
Low	57.78 ± 1.29 ^b^	60.10 ± 0.93 ^b^	73.87 ± 0.36 ^b^	87.46 ± 0.93	35.52 ± 1.19 ^b^
High	62.97 ± 1.61 ^a^	63.56 ± 0.70 ^a^	77.43 ± 0.36 ^a^	89.28 ± 0.72	41.27 ± 1.79 ^a^
*p*-Value					
Types of PKC	0.4510	0.1091	0.2056	0.0934	0.4572
Level of Oil	0.0200	0.0065	<0.0001	0.1284	0.0175
PKC × Oil	0.2413	0.8665	0.5931	0.8137	0.7549

^a, b^ Means within a column with no common letters differ at *p* < 0.05. PKC, palm kernel cake; LPKC, fermented PKC; Low, 5% oil; High, 9.5% oil; SE, standard error. DM, dry matter; GE, gross energy; CP, crude protein; EE, ether extract.

**Table 5:** Effect of type of PKC and level of oil on digestive enzyme activities in the intestinal contents (Mean ± SE).

Diet	Amylase, U	Protease, U	Lipase, U
Type of PKC			
PKC	10,256.60 ± 410.26	2233.00 ± 77.55	27.59 ± 1.10
LPKC	11,659.40 ± 466.38	2240.00 ± 96.32	26.58 ± 1.03
Level of Oil			
5.0%	13,485.90 ± 458.92 ^a^	2238.10 ± 70.24	25.90 ± 1.18
9.5%	8201.70 ± 354.10 ^b^	2233.20 ± 89.34	28.27 ± 0.86
*p*-Value			
Types of PKC	0.0586	0.9191	0.4974
Level of Oil	<0.0001	0.8876	0.1198
PKC × Oil	0.4964	0.5042	0.4469

^a, b^ Means within a column with no common letters differ at *p* < 0.05. PKC, palm kernel cake; LPKC, fermented PKC; Low, 5% oil; High, 9.5% oil; SE, standard error. One unit is the amount of amylase that cleaves ethylidene-pNP-G7 to generate 1.0 µmole of p-nitrophenol per minute at 25 °C. One unit is the amount of trypsin that cleaves the substrate, yielding 1.0 μmole of p-NA per minute at 25 °C. One unit is the amount of lipase that will generate 1.0 μmole of glycerol from triglycerides per minute at 37 °C.

**Table 6:** Intestinal villi height and crypt depth of broilers fed LPKC and PKC-based diets at different levels of oil (Mean ± SE).

Diet	Duodenum, µm		Jejunum, µm		Ileum, µm	
	Villi Height	Crypt Depth	Villi Height	Crypt Depth	Villi Height	Crypt Depth
Type of PKC						
PKC	1178.61 ± 42.50 ^b^	190.19 ± 7.00	1077.32 ± 24.79	146.85 ± 3.91 ^b^	804.54 ± 20.13	139.26 ± 4.57 ^b^
LPKC	1401.12 ± 54.51 ^a^	180.21 ± 4.49	1073.58 ± 31.69	166.30 ± 5.61 ^a^	803.95 ± 24.10	158.71 ± 6.04 ^a^
Level of Oil						
5%	1165.19 ± 45.17 ^b^	187.86 ± 6.70	1052.23 ± 26.02	157.29 ± 4.82	784.83 ± 19.66	148.92 ± 4.85
9.5%	1414.54 ± 48.11 ^a^	182.54 ± 5.16	1098.67 ± 29.50	155.86 ± 6.00	823.66 ± 23.44	149.04 ± 6.81
*p*-Value						
Type of PKC	0.0003	0.2482	0.9274	0.0103	0.9580	0.0193
Level of Oil	<0.0001	0.5348	0.2634	0.8418	0.2279	0.9883
PKC × Oil	0.7879	0.4416	0.8558	0.9212	0.5993	0.9410

^a, b^ Means within a column with no common letters differ at *p* < 0.05. PKC, palm kernel cake; LPKC, fermented PKC; Low, 5% oil; High, 9.5% oil; SE, standard error.

**Table 7:** Effects of feeding PKC and LPKC diets with different oil levels on mRNA expressions of SGLT-1, SGLT-5, Pep-T1, r-BAT, EAAT3, and FABP1 transporters (Mean ± SE).

Diet	mRNA Expression of Transporter, Folds Change					
	SGLT-1	SGLT-5	Pep-T1	r-BAT	EAAT3	FABP1
Types of PKC						
PKC	1.49 ± 0.06	1.40 ± 0.05 ^a^	0.46 ± 0.02	1.35 ± 0.07 ^b^	1.55 ± 0.07 ^b^	4.65 ± 0.18 ^a^
LPKC	1.38 ± 0.07	1.07 ± 0.06 ^b^	0.47 ± 0.03	1.79 ± 0.08 ^a^	2.04 ± 0.09 ^a^	3.39 ± 0.16 ^b^
Level of Oil						
Low	1.00 ± 0.03 ^b^	0.98 ± 0.04 ^b^	0.38 ± 0.01 ^b^	1.26 ± 0.05 ^b^	1.33 ± 0.07 ^b^	3.74 ± 0.22 ^b^
High	1.87 ± 0.06 ^a^	1.49 ± 0.04 ^a^	0.56 ± 0.02 ^a^	1.88 ± 0.07 ^a^	2.25 ± 0.11 ^a^	4.30 ± 0.19 ^a^
*p*-Value						
Types of PKC	0.0831	<0.0001	0.8092	<0.0001	<0.0001	<0.0001
Level of Oil	<0.0001	<0.0001	<0.0001	<0.0001	<0.0001	0.0159
PKC × Oil	0.0897	0.2219	0.5101	0.0897	0.2617	0.2404

^a, b^ Means within a column with no common letters differ at *p* < 0.05. PKC, palm kernel cake; LPKC, fermented PKC; Low, 5% oil; High, 9.5% oil; SE, standard error; SGLT-1, sodium-glucose transporter 1; SGLT-5, sodium-glucose transporter 5; Pep-T1, H+/peptide transporter; r-BAT, protein related to b^0,+^ amino acid transport system; EAAT3, excitatory amino acid transporter 3; FABP1, fatty acid-binding protein 1.

## Data Availability

Data sharing is not applicable to this article.
